# Overcoming Biases in Opportunistic Citizen Science for Studying Life History Traits of an Invasive Leaf-Mining Tree Insect Pest

**DOI:** 10.3390/insects16090929

**Published:** 2025-09-04

**Authors:** Natalia I. Kirichenko, Maria A. Ryazanova, Oksana V. Kosheleva, Stanislav Gomboc, Barbara Piškur, Maarten de Groot

**Affiliations:** 1 Slovenian Forestry Institute, Večna pot 2, 1000 Ljubljana, Slovenia; barbara.piskur@gozdis.si; 2 Sukachev Institute of Forest, Siberian Branch of the Russian Academy of Sciences, Federal Research Center “Krasnoyarsk Science Center SB RAS”, Akademgorodok 50/28, Krasnoyarsk 660036, Russia; rznv.m@mail.ru; 3 All-Russian Plant Quarantine Center (VNIIKR), Pogranichnaya Str. 32, Bykovo, Moscow 140150, Russia; 4 Institute of Ecology and Geography, Siberian Federal University, Svobodny pr. 79, Krasnoyarsk 660041, Russia; 5 All-Russian Institute of Plant Protection (FSBSI VIZR), Podbelskogo 3, Saint Petersburg 196608, Russia; koscheleva_o@mail.ru; 6 Independent Researcher, Gančani 110, 9231 Beltinci, Slovenia; stanislav.gomboc@siol.net

**Keywords:** iNaturalist, invasive leaf miner, *Macrosaccus robiniella*, leaf mine types, Europe, North America

## Abstract

The aim of this study was to determine whether opportunistic citizen science can support the detection of life history traits in invasive insects. Using the invasive leaf-mining micromoth *Macrosaccus robiniella* (Clemens 1859) (Lepidoptera: Gracillariidae) as a model species, we analyzed data from iNaturalist submitted by citizen scientists to assess the variability in its leaf mines on its native host, *Robinia pseudoacacia* L., 1753 (Fabaceae), across both the moth’s invaded (Europe, North America–Eastern United States) and native range (North America–Southern and Western Unites States, Eastern Canada). We examined 86,489 photographs collected over the past 20 years to compare the occurrence and proportions of different *M. robiniella* leaf mine types between invaded and native ranges using three search variants: (I) *M. robiniella*, (II) all endophagous invasive insects associated with *R. pseudoacacia*, and (III) the host plant itself. The first two datasets revealed differences in the ratio of leaf mine types between Europe and North America (when analyzed separately for native and invaded areas), whereas the third dataset showed no significant differences in either the presence or proportion of mine types between invaded and native ranges. Leaf mine types atypical of *M. robiniella*, which resemble damage caused by other invasive insects such as *Parectopa robiniella* Clemens, 1863 (Lepidoptera: Gracillariidae) and *Obolodiplosis robiniae* (Haldeman, 1847) (Diptera: Cecidomyiidae)—also associated with *R. pseudoacacia*—have been observed in Europe for at least a decade. Our main conclusion is that, when investigating the life history traits of invasive herbivorous insects, focusing data collection on the host plant rather than on the insect species alone can reduce biases associated with opportunistic citizen science and help reveal true ecological patterns.

## Simple Summary

Photos shared by volunteers on the biodiversity platform iNaturalist offer valuable insights into invasive tree insect ecology but require careful extraction methods. We studied *Macrosaccus robiniella*, a leaf-mining moth, comparing its damage patterns (leaf mines) between invaded regions (Europe, North America–Eastern United States) and its native habitat (other parts of North America–Southern and Western Unites States, Eastern Canada). Analyzing 86,489 volunteer-collected images over two decades revealed three search strategies: directly searching *M. robiniella* (I), related invasive pests on *Robinia pseudoacacia* trees (II), and targeting the tree itself (III). Search strategy III, focused on the host plant, yielded critical findings. Contrary to previous knowledge suggesting only lower-side leaf-mining in Europe, diverse mine types were discovered in the invaded range via this method. These results highlight the effectiveness of prioritizing searches based on host plants rather than specific pest species when investigating biology of invasive tree insects.

## 1. Introduction

Citizen science has become an important tool in invasion science, particularly in the study of invasive alien insects [1,2,3,4]. By engaging volunteers, nature observers, and amateur scientists, citizen science enables large-scale data collection, facilitating the monitoring of species distributions, population dynamics, and ecological interactions across vast geographical areas and extended timeframes [5,6,7,8,9].

The quality and reliability of data collected through citizen science depends on multiple factors, including the training level of participants and the methods used. Structured citizen science projects, in which volunteers follow standardized sampling protocols, have been shown to generate high-quality data [9,10]. Such data are valuable for tracking species spread, identifying biodiversity trends, and detecting novel ecological interactions [6,11]. Conversely, opportunistic citizen science based on records collected by untrained nature observers and incidental documentation, may introduce biases due to misidentifications, selective reporting, or reliance on outdated knowledge [12,13,14,15]. These biases can limit the completeness of datasets and affect the conclusions drawn. Nonetheless, even opportunistic data, when analyzed critically, can be used to explore ecological patterns and provide valuable insights into the characteristics of invasive species.

In this study, we investigate whether opportunistic citizen science data can provide reliable information on the life history traits of invasive insects in their introduced range, and how potential observer biases can be mitigated. As a model species, we used the leaf-mining moth *Macrosaccus robiniella* (Lepidoptera: Gracillariidae). This species was originally described under the genus *Lithocolletis* Hübner, 1825, later reassigned to *Phyllonorycter* Hübner, 1822, and, just over a decade ago, was transferred to the newly established genus *Macrosaccus* Davis & De Prins, 2011 [16].

*Macrosaccus robiniella* is a North American leaf-mining moth associated with black locust (*Robinia pseudoacacia*), a tree species widely planted outside its native range for ornamental landscaping, forestry, and honey production. While black locust was introduced to Europe in the second half of the 17th century [17,18], *M. robiniella* was first recorded on the continent much later in Switzerland in 1983 [19]. Since then, the moth has spread across much of Europe [20,21]. Three other endophagous insect species of North American origin have also colonized black locust in Europe: the leaf-mining moth *Parectopa robiniella*, the gall-forming midge *Obolodiplosis robiniae*, and the leaf-mining moth *Chrysaster ostensackenella* (Fitch, 1859) (Lepidoptera: Gracillariidae), which was most recently detected (so far only in Italy) [22,23,24,25].

In Europe, *M. robiniella, P. robiniella*, and *O. robiniae* coexist within the same habitats, utilize the same host trees, and in some instances feed on the same leaflets [23,25]. Each species, however, exhibits distinct feeding patterns: *M. robiniella* makes lover-side oval blotch leaf mines, *P. robiniella* creates upper-side mines along the midrib [19,22,26,27,28,29], and *O. robiniae* induces leaf-margin galls that cause leaflet folding [26]. Such differentiation allows these three endophagous foliar feeders to occupy slightly distinct niches while exploiting the same host plant [23].

In Europe, *M. robiniella* has traditionally been known by the lower-side oval mines [19,22,26,27,28,29]. A single occurrence of an upper-side mine was documented in Switzerland—specifically, upper-side oval mines located between the main rib and the leaf edge [19]. In the moth’s native range in the USA, variation in the positions of *M. robiniella* leaf mines was documented early on, with mines on the upper side and at the lower leaf margin considered atypical and less abundant [30]. In 2023, we observed atypical *M. robiniella* mines on *R. pseudoacacia* in Slovenia and Croatia that had not been previously documented in the European literature, despite the invasive moth being well-studied on the continent. These included upper-side leaf mines situated above the midrib, somewhat resembling those of *P. robiniella*, and lower-side edge-situated mines causing downward leaflet folding, a pattern typically associated with *O. robiniae.* Surprisingly, the relative abundance of these atypical mine types was about four times higher than that of the classical lower-side blotch mines [8]. The absence of earlier records describing these mine types in Europe, where the insect is well-studied, led us to hypothesize that over time since its introduction, *M. robiniella* may have progressively exhibited the variability and abundance in feeding behavior similar to that observed in its native range [30]. Furthermore, parasitism rates in the atypical mines (i.e., *Parectopa*-like and *Obolodiplosis*-like mines) appeared to be lower than those in the classical lower-side mines, suggesting potential ecological advantages [8].

To investigate whether these life history traits are gradually exhibited in *M. robiniella* in its invaded range in Europe, we used opportunistic citizen science data. Specifically, we analyzed all photographic records of *M. robiniella* leaf mines posted on the crowdsourcing platform iNaturalist (https://www.inaturalist.org/, accessed on 3 September 2025) over the last two decades. We hypothesized that using records retrieved by species name may introduce bias, as untrained citizen scientists tend to document familiar or well-known features, potentially overlooking novel or emerging traits. In contrast, records retrieved by the host plant name were hypothesized to provide a broader and potentially less biased dataset, as the main focus in host plant photos is the host plant itself rather than leaf damage. At the same time, it was expected that some images posted under the host plant name might feature close-up views of leaves displaying various types of insect damage.

We used a structured approach to validate our findings. We compiled and analyzed three types of datasets representing *M. robiniella* mine occurrence, extracted using the following search strategies: (1) the name of *M. robiniella*, (2) the names of all endophagous invasive insects associated with *R. pseudoacacia* in Europe, and (3) the host plant name, *R. pseudoacacia.* By comparing these datasets, we aimed to determine whether observed trends reflect a genuine biological phenomenon or are an artifact of data collection biases. Specifically, we determined (1) whether unusual mine types observed in European countries have emerged recently in the moth’s invasive range and (2) whether similar feeding patterns are present in the moth’s invaded and native ranges in North America.

## 2. Materials and Methods

### 2.1. Retrieving Data from a Citizen Science Platform

We analyzed photographs of endophagous insect damage on *R. pseudoacacia* leaves, captured through opportunistic citizen observations and posted on [31]. These images span the past 20 years (2005–2024) and originate from 39 U.S. states and 6 Canadian provinces in North America, as well as 22 countries in Europe (Figure S1).

In North America, *R. pseudoacacia* is native to a restricted area within the central and eastern United States [32]. Thus, the moth’s native range includes Kentucky, Missouri, Ohio, Pennsylvania, Virginia, and West Virginia, while its presence in other U.S. states and in Canada—where *R. pseudoacacia* was introduced—represents its invaded range.

**Figure 1:**
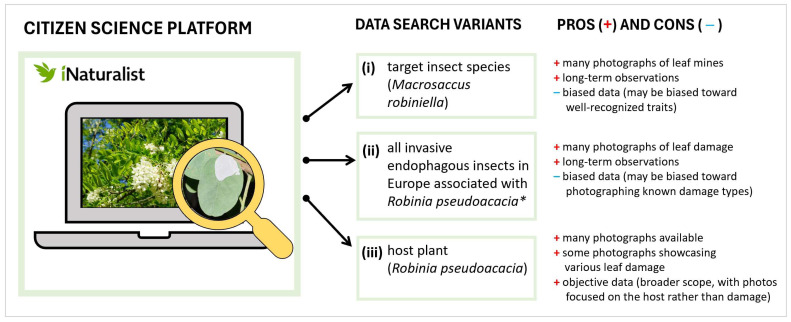
Variants of data search used on the iNaturalist platform. * This includes four species: three leaf-mining species (*Macrosaccus robiniella, Parectopa robiniella, Chrysaster ostensackenella*) and one leaf-galling species (*Obolodiplosis robiniae*).

Photographs were retrieved using three variants: (I) images posted under the name of *M. robiniella;* (II) images posted under the names of endophagous foliar insects on *R. pseudoacacia*, invasive in Europe, including leaf miners *P. robiniella* and *Ch. ostensackenella*, and leaf galler *O. robiniae*, in addition to images posted under the name *M. robiniella;* and (III) images posted under the name of the host plant, *R. pseudoacacia* (Figure 1; Table S1).

Search variant II was intended to expand upon variant I by incorporating records where leaf mines of *M. robiniella* might have been misidentified by untrained citizen scientists and attributed to the other invasive endophages mentioned above. We also assumed that *M. robiniella* mines could have been unintentionally photographed alongside damage caused by other endophagous insects.

We hypothesized that search variants I and II might introduce bias, as the photographs posted under the names of certain insects might be focused on known or typical damage patterns. In contrast, search variant III was expected to yield a broader and potentially less biased dataset, as it centered on the host plant itself rather than leaf damage. At the same time, we recognized that some images posted under the name of *R. pseudoacacia* might feature close-up views of leaves displaying various types of insect damage.

When examining damage caused by *M. robiniella*, four mine types were taken into account (i.e., mine types 1–4): (1) lower-side blotch mine (i.e., oval tentiform blotch situated on the lower side of the leaflet on one of the leaflet halves), or mine type 1; (2) upper-side blotch mine (i.e., a blotch occupying one half of a leaflet on its upper side), or mine type 2; (3) upper-side mine above midrib (*Parectopa*-like mine) (i.e., an elongated blotch located on the upper side of the leaflet above the midrib, causing upward folding of the leaflet in the middle), or mine type 3; (4) lower-side edge mine, or *Obolodiplosis*-like mine (i.e., an elongated blotch situated on the lower side of leaflet next to the leaflet edge, causing strong downward folding), or mine type 4. These mine types are described and illustrated in our previous study [8].

In total, 86,489 photographs were manually analyzed by three authors (NIK, KOV, RMA) with experience in identifying leafminer damage. Each photo was reviewed to verify whether the observed damage could be confidently attributed to *M. robiniella* and to assign the observed mines to one of the four predefined mine types. If more than one mine type appeared in a single photo, each type was counted as a separate incident. Thus, the number of incidents, i.e., the presence of a certain mine type of *M. robiniella* in a given photograph, was counted. As a result, three datasets were compiled corresponding to the three search variants. For each incident, the dataset included the country, the classification as native or invaded range, the year the photo was taken, and the type of *M. robiniella* mine present.

### 2.2. Data Analysis

The number of photographs documenting *M. robiniella* mines was analyzed for each dataset and expressed as a percentage relative to the total number of photographs posted under species names across the three search variants. The number of incidents of various *M. robiniella* mine types appearing in photographs was assessed for each search variant and expressed as a percentage of the total photographs depicting *M. robiniella* mines.

The datasets containing information on the presence of different *M. robiniella* mine types generated from the three search variants were analyzed separately. Data between serach variants and within each variant for the native and invaded ranges were examined using Chi-square test with a Holm’s correction [33]. For the invaded range, data from Europe and North America, where the insect is non-native, were analyzed separately.

The number of records and mine type occurrences from 2005 to 2024 was estimated to identify the earliest appearance of each mine type in Europe. To illustrate changes over time, the annual ratio of *M. robiniella* mine types was plotted to show their dynamics in both the native and invaded ranges.

## 3. Results

### 3.1. Number of Records of M. robiniella Mines Detected on iNaturalist

**Figure 2:**
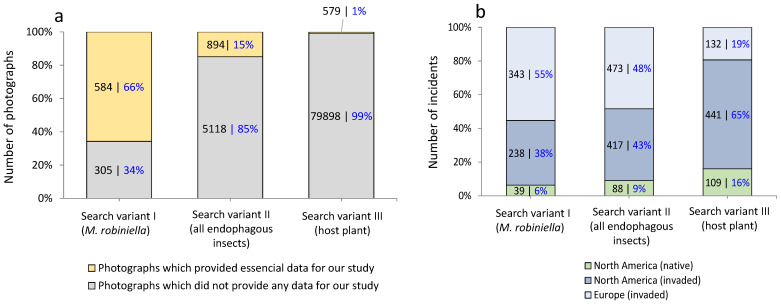
Total number of photographs (expressed in %) analyzed across different search variants (**a**) and number of incidents (i.e., the presence of a certain mine type of *M. robiniella* in photographs) per studied region in different search variants (**b**). In (**a**), photographs surveyed in search variant I were also included in search variant II, where all invasive endophagous insects associated with *Robinia pseudoacacia* were considered, including *M. robiniella*. Consequently, the total number of photographs providing data for our study was 1473 (i.e., 894 + 579), while 85,016 (i.e., 51,118 + 79,898) photographs yielded no relevant data out of 86,489 photographs (i.e., 1473 + 85,016) examined in total. Above bars, *p*-values are provided (Chi-square test).

Among the 86,489 photographs analyzed on iNaturalist across the three search variants (I—*M. robiniella*, II—all endophagous insects, and III—host plant), only 1473 photographs (about 2%) contained data on *M. robiniella* leaf mines. The number of photographs providing important data for our study varied across the three search variants. Search variant I (*M. robiniella*) yielded 66% of photographs (584 out of 889) containing identifiable *M. robiniella* leaf mines (Figure 2a). The remaining 34% of photographs in this variant showed *M. robiniella* adults and thus could not be used in our study.

In search variant II (all endophagous insects), the proportion of the photographs containing needed data for the study dropped to 15% (894 out of 6012 photographs). The remaining photographs (85%) showed leaf mines of different endophagous species feeding on Robinia, but not *M. robiniella*, or illustrated their adults. Search variant III (host plant) yielded the lowest percentage, with only about 1% of photographs (579 out of 80,478) containing relevant data (Figure 2a). The remaining 99% of photographs showed the whole tree, some parts of its crown or its branches, not useable for our study. Values in search variants I-III were statistically different, *p* < 0.05 (Chi-test) (Figure 2a,b).

Overall, 620 incidents of various *M. robiniella* mine types were recorded in 584 photographs depicting leaf damage caused by this species in search variant I, contributing an additional 6% of data to the dataset. In search variant II, 978 incidents were identified in 894 photographs, adding 9% more data. In search variant III, 682 incidents were extracted from 579 photographs, increasing the dataset by 18%.

The number of *M. robiniella* mine incidents was higher in its invaded range (Europe and part of North America) than in its native range (other regions of North America), which accounted for only 6% to 16% of all records (Figure 2b).

### 3.2. Occurrence of M. robiniella Mine Types in Invaded vs. Native Ranges

**Figure 3:**
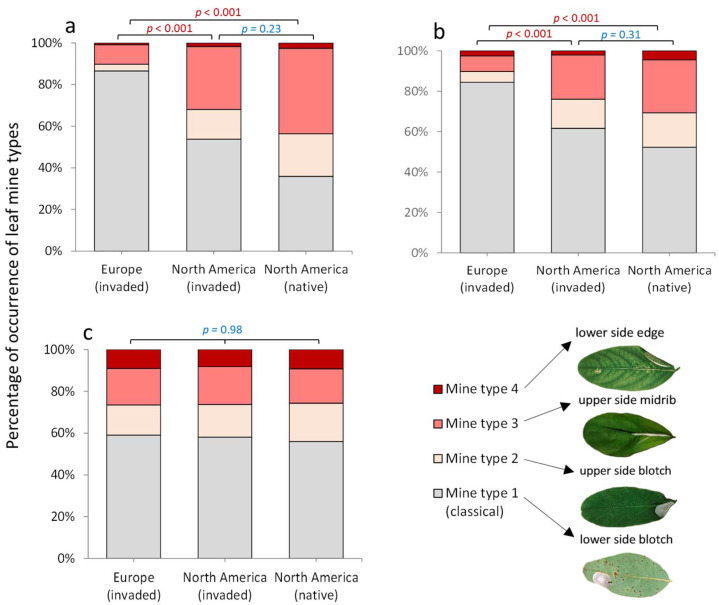
Percentage of occurrence of *M. robiniella* mine types in invaded and native ranges, based on public data for (**a**) *M. robiniella*, (**b**) all invasive endophagous insects associated in Europe with *R. pseudoacacia*, and (**c**) the host plant. Above bars, *p*-values are provided (Chi-square test).

The analysis of search variants I and II revealed a significant difference in the proportion of *M. robiniella* mine types between Europe (invaded range) and North America (native and invaded ranges) (Figure 3a,b). However, no statistical difference was found for search variant III (Figure 3c).

Across all studied geographic regions, all four mine types were recorded, with mine type 1 (classical mine) being the most predominant (Figure 3a–c). In Europe, under search variants I and II, the proportion of mine type 1 was considerably higher than in North America (both native and invaded ranges) (Figure 3a,b).

### 3.3. Temporal Trends in Documenting Mine Types Across Native and Invaded Ranges

**Figure 4:**
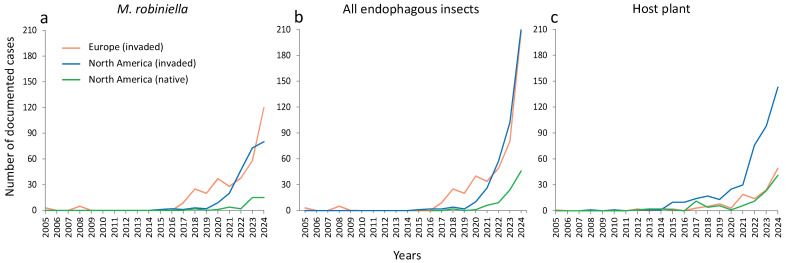
Number of *M. robiniella* mine incidents revealed in photographs posted on the iNaturalist platform over the last 20 years (2005–2024) based on three search variants: (**a**) *M. robiniella*, (**b**) all invasive endophagous insects associated in Europe with *R. pseudoacacia*, and (**c**) the host plant, *R. pseudoacacia*.

The number of *M. robiniella* mine incidents extracted from photographs submitted by opportunistic citizen scientists increased sharply over time across all search variants and in both native and invaded ranges (Figure 4). Notably, more than 50% of all records were documented in the period 2023–2024.

**Figure 5:**
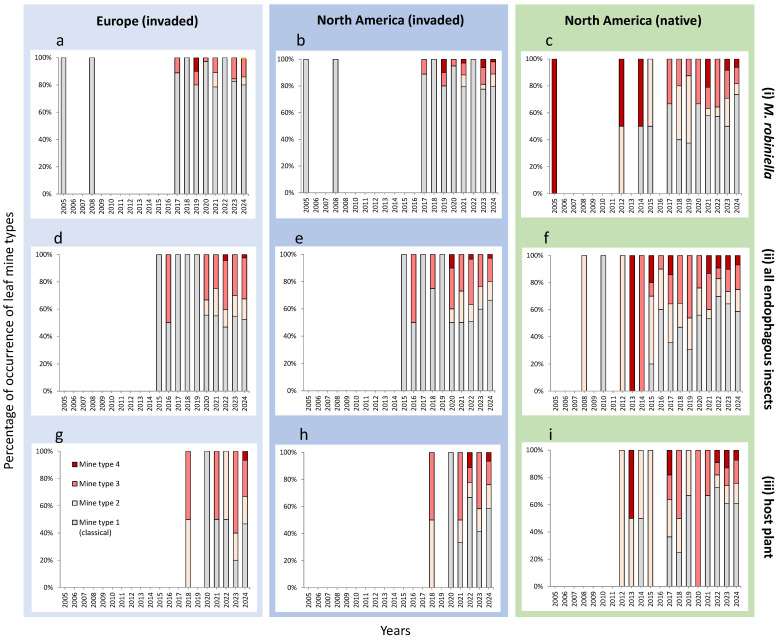
Percentage of occurrence of *M. robiniella* mine types in the invaded vs. native ranges in the period 2005–2024, based on public data for (**a**–**c**) *M. robiniella*, (**d**–**f**) all invasive endophagous insects associated with *Robinia pseudoacacia* in Europe, and (**g**–**i**) the host plant (*R. pseudoacacia*).

No clear temporal patterns were observed in the proportion of different *M. robiniella* mine types in Europe and North America over the study period (2005–2024) across all three search variants (Figure 5). Mine types 2 and 3 were detected on both continents throughout the study period (Figure 5).

In the invaded ranges of Europe and North America, these atypical mines have been documented since 2016, although the classical mine type (type 1) remained predominant (Figure 5d,e). In the native range of North America, leaf mines of different types were more evenly distributed across all search variants, but no distinct temporal pattern was revealed over the study period (Figure 5c,f,i).

## 4. Discussion

### 4.1. Using Opportunistic Citizen Science Data to Study Invasive Insect Traits

Data from crowdsourced platforms is of great importance for studying invasive insects, as volunteers can often spot and opportunistically photograph characteristic damage or the insects themselves well before researchers and practitioners detect them in a region [34,35,36]. As we have shown, such opportunistic data can also be highly valuable for investigating the biological traits of invasive insects, particularly foliar species. In this sense, our study provides one of the pioneering examples of how to deal with these data to obtain reliable information on the biological characteristics of leaf-mining invasive insects in invaded range vs. their native range.

Using the invasive micromoth *M. robiniella* as an example and analyzing a large opportunistic citizen science dataset (86,489 photographs), we were able to track the presence and relative proportions of different mine types on *R. pseudoacacia* leaves in both native and invaded ranges. In particular, we showed that of the three search strategies applied on iNaturalist, search variant I (*M. robiniella*) was most effective, yielding 66% relevant photographs in both ranges (579 out of 889). Search variant II (all invasive endophagous insects) added one-third more records but did not reduce bias. In contrast, search variant III (host plant) required analyzing a much larger dataset (80,579 photos), yet produced less biased results and a comparable number of relevant records (584). Despite the effort, variant III provided richer information on leaf mine diversity and 18% more data overall, whereas variants I and II contributed only 6% and 9% additional information, respectively.

Therefore, the analysis of photographs posted under the host plant name did not support the hypothesis that *M. robiniella* has only recently exhibited variable feeding behaviors in its invasive range. Specifically, the presence of atypical mine types in Europe, resembling damage caused by other invasive *Robinia* pests such as *P. robiniella* and *O. robiniae*, has been documented by opportunistic observers for over a decade. Notably, all leaf mine types recorded in our study via iNaturalist in Europe (the invaded range) had previously been documented in the native range of *M. robiniella* (eastern United States) [30].

Thus, the similarity in the distribution of different mine types between native and invaded ranges further suggests that these atypical mine types are a pre-existing trait of *M. robiniella.* Rather than reflecting new evolutionary changes, the invaded range may simply provide more opportunities for these behaviors to be observed. These findings support the idea that invasive species may exploit behaviors already present in their native range, rather than developing entirely new ones after invasion [37,38].

### 4.2. Data Verification from Crowdsourced Platforms

While citizen science platforms that store opportunistic records offer a wealth of data, it is important to consider potential biases and uncertainties arising from the observer effect [39,40]. Such data are subjective because data sampling does not follow any specific protocols, thus, they may include misidentifications, selective reporting, underreporting, and other biases inherent to crowdsourcing platforms [15], making it challenging to draw reliable conclusions about invasive insect species [3]. However, as demonstrated in our study, a critical approach to opportunistic data can help mitigate these concerns, allowing opportunistic citizen science to remain a valuable tool for studying the life history traits of invasive insects across large spatial and temporal scales.

In our case study, we implemented a multi-step validation process to ensure the reliability of the records included in the analyses. Specifically, we validated (i) the assignment of leaf damage to different endophagous species and (ii) the identification of *M. robiniella* leaf mines and their classification into four defined mine types. The initial data survey on iNaturalist was manually conducted by trained researchers (the manuscript authors), and the final validation of data classification was performed by the coauthor specializing in leaf miners (NIK). This procedure allowed for rigorous data filtering and ensured that only validated records were used in subsequent analyses. Furthermore, as a reference group, the involvement of search variant III—host plant (*R. pseudoacacia*)—produced the most objective data on the diversity of leaf mine types compared with searches targeting only endophagous invasive insects (including *M. robiniella* and other invasive *Robinia*-feeding species sharing the same trophic resource). And we clearly demonstrated that this strategy provided a more reliable baseline and helped evaluate potential biases in the dataset.

While our study did not aim to validate iNaturalist data in a general sense, our approach effectively demonstrates how opportunistic citizen science data can be meaningfully validated and reliably used to detect ecological patterns in invasive leaf miner when expert knowledge is integrated into the data extraction and analysis process.

### 4.3. Limitations

Our study has several limitations regarding the use of crowdsourced platforms for research on biological traits of invasive phytophagous insects. First, our study approach is applicable only to invasive endophagous foliar insects, such as leaf-mining and leaf-galling species, whose damage remains on leaves throughout the season, allowing identification of the insect species even after they have left their shelters.

Second, we manually examined a large number of photographs posted on iNaturalist, a routine yet time-consuming task. This step was essential to address the variability in image quality and relevance. While Machine Learning (ML) holds promise for automating image analysis [41,42], its application in this context is currently limited. Images uploaded by untrained citizen scientists often lack the consistency and clarity required for reliable automated processing. ML models require extensive training and validation on diverse, high-quality datasets to accurately identify key features such as leaf mines.

The third limitation was the low percentage of useful photographs among the large number analyzed on iNaturalist. This issue was particularly evident when examining the images of the host plant, where less than 1% of the images provided relevant data. This highlights the considerable effort required to extract reliable information from crowdsourced platforms.

Finally, the temporal coverage of the photographs on iNaturalist was incomplete. Not all years were equally represented, and the overall dataset spans only two decades (i.e., 2005–2024). This limited coverage hindered our ability to detect long-term trends or to conduct more detailed temporal analyses of life history evolution in invasive insects. Because citizen scientists have only recently increased their engagement with such platforms, the data is skewed toward more recent years, limiting our ability to draw broader conclusions about long-term changes in life history traits.

### 4.4. Future Perspectives

A further potential use of opportunistic citizen science data lies in applying ML and artificial intelligence (AI) to extract relevant information from crowdsourced platforms. This would greatly reduce the time required for analysis—unlike in our case, where manual examinations of more than 80,000 iNaturalist images took several months. Indeed, ML and AI are increasingly applied in ecological studies [43,44], and their use will undoubtedly expand, becoming more accurate in identifying insect pests [45,46] and distinguishing insect damage patterns and linking them, whenever possible, to particular species [47].

To increase useful data volume and quality, invasion researchers should proactively post early-warning materials (photos of diagnostic damage, host lists, seasonal cues) and calls-to-action on citizen science platforms, which will help mobilize volunteers to document these species and their host plant damage [15,36]. Over time, such contributions will provide invaluable resources for deeper ecological and invasion biology studies [34,48].

## 5. Conclusions

Our study highlights the potential of opportunistic citizen science data as a valuable resource for investigating life history traits in invasive endophagous (in particularly, leaf-mining and leaf-galling) insect species. However, such data must be carefully evaluated on a case-by-case basis to avoid potential biases. While observations from untrained contributors may introduce inconsistencies, thoughtful analysis can still reveal important ecological patterns that might otherwise go unnoticed or be misinterpreted. Using the leaf-mining moth *M. robiniella* as an example, we clearly demonstrated that data searches on crowdsourcing platforms should focus primarily on the host plant (i.e., search variant III as applied in our study) rather than on the insect species (i.e., search variants I and II), in order to minimize potential biases. Through this approach, we discovered that the occurrence of alternative mine types in Europe had been previously overlooked. By leveraging long-term observations contributed by nature enthusiasts, we were able to identify these atypical mines in both the native and invaded ranges of the species. Future studies should continue to explore the application of opportunistic citizen science data in studying life history adaptations of invasive species within a broader ecological context.

## Data Availability

All data supporting the findings of this study are available within the paper and its Supplementary Information (Table S1, Figure S1).

## Supplementary Materials

The following supporting information can be downloaded at https://www.mdpi.com/article/10.3390/insects16090929/s1, Table S1: Number of incidents, i.e., occurrence of a specific *Macrosaccus robiniella* mine type in photographs, retrieved from iNaturalist using three search variants: (I) images posted under the name *M. robiniella*; (II) images posted under the names of endophagous foliar insects that are invasive in Europe (i.e., *M. robiniella*, *Parectopa robiniella*, *Chrysaster ostensackenella*, and *Obolodiplosis robiniae*); and (III) images posted under the name of the host plant, *Robinia pseudoacacia*; Figure S1: Number of recorded incidents (i.e., occurrences of a specific *M. robiniella* mine type in photographs) across its native and invaded ranges in the Northern Hemisphere.
