# Supplementary material for: Overcoming Biases in Opportunistic Citizen Science for Studying Life History Traits of an Invasive Leaf-Mining Tree Insect Pest
Source: Insects. 2025 Sep 4;16(9):929. doi: 10.3390/insects16090929 (PMC12470456; doi:10.3390/insects16090929)
Supplement: Supplementary file 1 [file insects-16-00929-s001.zip › Figure S1.pdf]

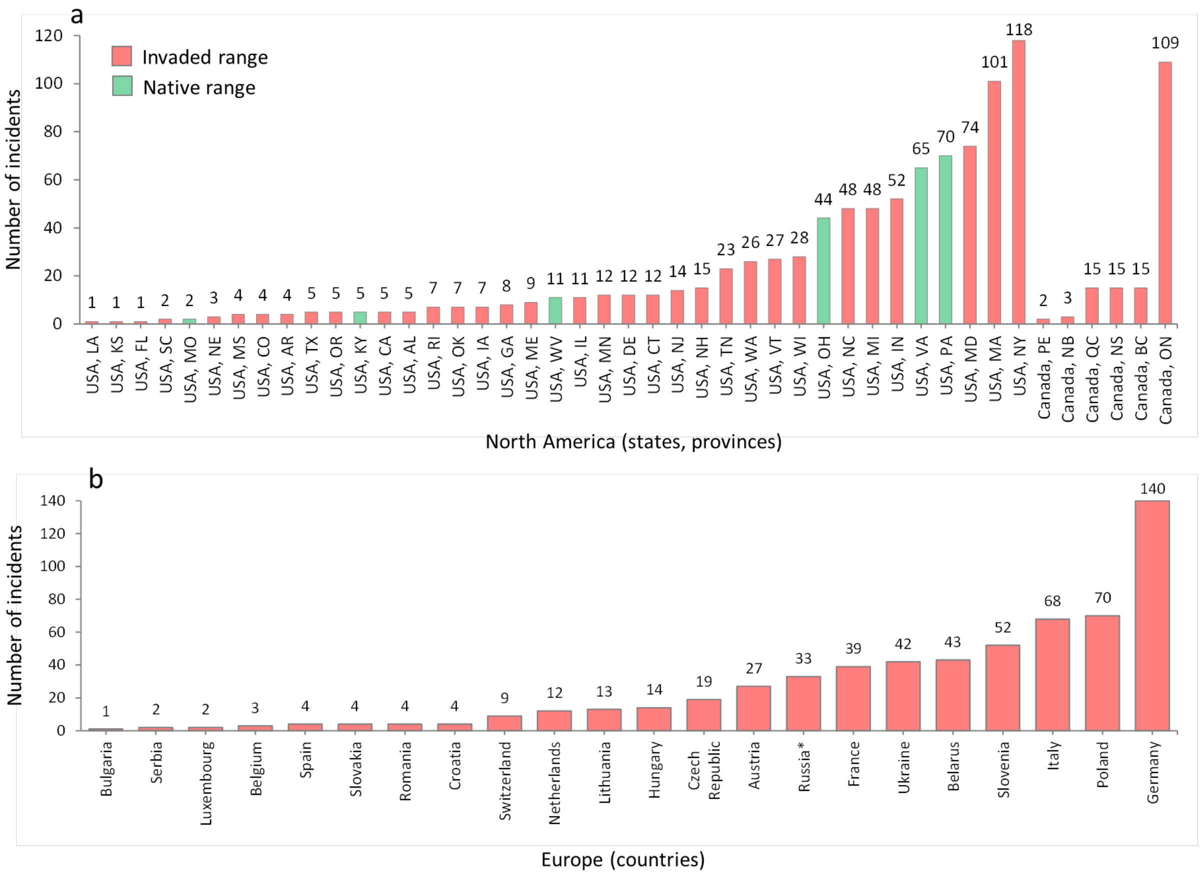

Figure S1. Number of recorded incidents (i.e., occurrences of a specific *M. robiniella* mine type in photographs) across its native and invaded ranges in the Northern Hemisphere. USA states: AL – Alabama, CA – California, CO – Colorado, CT – Connecticut, DE – Delaware, FL – Florida, GA – Georgia, IA – Iowa, IL – Illinois, IN – Indiana, KS – Kansas, KY – Kentucky, LA – Louisiana, MA – Massachusetts, MD – Maryland, ME – Maine, MI – Michigan, MN – Minnesota, MO – Missouri, MS – Mississippi, NC – North Carolina, NE – Nebraska, NH – New Hampshire, NJ – New Jersey, NY – New York, OH – Ohio, OK – Oklahoma, OR – Oregon, PA – Pennsylvania, RI – Rhode Island, SC – South Carolina, TN – Tennessee, TX – Texas, VA – Virginia, VT – Vermont, WA – Washington, WI – Wisconsin, WV – West Virginia; Canadian provinces: BC – British Columbia, NB – New Brunswick, NS – Nova Scotia, ON – Ontario, PE – Prince Edward Island, QC – Quebec. *Russia – European part only.
